# Peer learning in clinical placements in psychiatry for undergraduate nursing students: preceptors and students’ perspective

**DOI:** 10.1002/nop2.602

**Published:** 2020-08-18

**Authors:** Verica Vuckovic, Kajsa Landgren

**Affiliations:** ^1^ Psychiatric Clinic in Helsingborg Office of Psychiatry and Habilitation Region Skåne Sweden; ^2^ Department of Health Sciences Faculty of Medicine Lund University Lund Sweden; ^3^ Psychiatric Clinic in Lund Office of Psychiatry and Habilitation Lund Sweden

**Keywords:** clinical placements, nurse student, peer learning, preceptor, psychiatric nursing, qualitative, questionnaire

## Abstract

**Aim:**

To describe the experiences of peer learning in psychiatric inpatient settings during clinical placement of undergraduate nursing students and to highlight the possibility for peer learning in psychiatric outpatient settings.

**Design:**

A qualitative inductive design.

**Method:**

Questionnaires with 14 students and 12 preceptors in inpatient and outpatient care and interviews with one student and one preceptor in outpatient care were analysed with content analysis.

**Results:**

Students and preceptors perceived learning benefits with peer learning. They described how learning increased through exchange of knowledge and how collaboration created security and independence, structured learning activities were appreciated as a learning tool. Incompatibility of students was an issue that could be overcome. Peer learning was perceived to contribute to a secure learning atmosphere, increased self‐confidence and to provide a deeper understanding of psychiatric nursing. Peer learning was described as promoting discussion and reflection on practice and preparing nursing students for their future profession.

## 1 INTRODUCTION

Clinical placements are a vital part of nursing education. A growing number of nursing students combined with fewer numbers of clinical placements lead to a need for new pedagogical models in nurses’ clinical practice. Peer learning (PL) is a learning model where two students collaborate and learn with and from each other while they reflect and solve problems together, guided by a preceptor (Pålsson, 2020). PL has been introduced and found to be a valuable pedagogical model in medical and surgical hospital wards (Hellström‐Hyson, Mårtensson, & Kristofferzon, 2012; Mamhidir, Kristofferzon, Hellström‐Hyson, Persson, & Mårtensson, 2014; Nygren & Carlson, 2017; Stenberg & Carlson, 2015, Pålsson et al., 2017). There is a gap of knowledge about PL in psychiatric in‐ and outpatient clinical placements. This article contributes to further understanding of the possibilities and drawbacks with PL in a psychiatric context.

## 2 BACKGROUND

Classroom education and practicing techniques in skills laboratories are crucial to students’ development, but experience in real clinical settings is invaluable for preparing students for their future profession (Budgen & Gamroth, 2008; Happell, Gaskin, Byrne, Welch, & Gellion, 2015; Jeppesen, Christiansen, & Frederiksen, 2017, Pålsson et al., 2017). During the clinical placements, the clinical nurse plays a crucial role in developing students’ learning (Budgen & Gamroth, 2008; Löfmark, Thorkildsen, Råholm, & Natvig, 2012; Mamhidir et al., 2014). The preceptorship model (PM) means that the student is paired with a professional nurse responsible for the teaching, communication and sharing of the practical realties (Young et al., 2007). Benefits of PM are that it provides the student with an expert preceptor in the clinical area, role socialization and opportunities to participate in patient care (Budgen & Gamroth, 2008). The limitations of PM are that the preceptor may not be an expert in teaching, it can mean an added workload for the preceptor and the student, and preceptor may be an incompatible match (Budgen & Gamroth, 2008).

More recently, peer learning (PL) has been introduced as an educational model for learning and teaching during nursing students' clinical placements. PL is defined as learning from and with each other, both in formal and informal ways (Boud, Cohen, & Sampson, 2016). PL emphasizes the learning process, entailing both the emotional support that learners provide to one another and the learning task itself. PL differs from PM as students learning takes place without immediate intervention by the teacher or the preceptor (Topping, 2005), which means other conditions and opportunities for preceptorship. PL in clinical placements encompasses students who are assigned the same clinical placement, work and reflect together and learn from each other supported by their joint preceptor. PL promotes lifelong learning outcomes, such as working together with others, social interaction, organizing learning and moving from dependence to independence, critical inquiry and reflection, communication and articulation of knowledge, and self‐ and peer assessment (Boud et al., 2016; Pålsson, 2020). Advantages of PL, according to nursing students in somatic care, were practical and emotional support from the peer, feeling secure, increased confidence, self‐efficacy and competence, and decreased anxiety (Chojecki et al., 2010; Christiansen & Bell, 2010; Hellström‐Hyson et al., 2012; Ravanipour, Bahreini, & Ravanipour, 2015; Stenberg & Carlson, 2015; Stone, Cooper, & Cant, 2013, Pålsson et al., 2017). Preceptors in somatic care perceived that PL contributed to active and independent nursing students who turned to each other first to solve nursing tasks before engaging the preceptor; (Mamhidir et al., 2014; Nygren & Carlson, 2017). In a study comparing PL and PM, students working with a peer rated collaboration and problem‐solving ability and reflection higher than students without a peer (Ekstedt, Lindblad, & Löfmark, 2019). However, some challenges with PL have been recognized. Incompatible students and the competition to perform nursing tasks and getting the preceptors’ attention could create difficulties for both students and preceptors (Nygren & Carlson, 2017; Stenberg & Carlson, 2015, Pålsson et al., 2017). Besides better learning outcomes, a reason for implementing PL could be a shortage of clinical placements (Stenberg & Carlson, 2015).

There is a gap of knowledge about PL in psychiatric settings. To our knowledge, there is only one published article on PL in a psychiatric context (Vuckovic, Karlsson, & Sunnqvist, 2019), a focus group study with preceptors and nurse students finding that PL promoted learning also in this context, in line with evaluations of PL in somatic care. Psychiatric settings differ from somatic settings. Psychiatric nursing focuses less on medical–technical tasks but emphasizes on creating a nurse–patient relationship, which can be challenging for nursing students (Demir & Ercan, 2018; Ejneborn Looi et al., 2016). Prior to clinical practice, nursing students feared caring for psychiatric patients and worried about their own personal safety due to potentially unpredictable or violent behaviours of psychiatric patients (Al‐Zayyat & Al‐Gamal, 2014; Ganzer & Zauderer, 2013; Webster, 2009; Wedgeworth, Ford, & Tice, 2020). Communication and interaction with patients in psychiatric settings during clinical placements could reduce students’ anxiety and fear, increase their confidence and increase their desire to work in psychiatric care (Happell et al., 2015; Moxham et al., 2015; Thongpriwan et al., 2015). The benefits of PL in somatic clinical placements described in previous research could possibly be transferred to psychiatric clinical placements and not only facilitate students` learning in psychiatric nursing but also ease their stress.

The nurse's possibilities to act as preceptor for students differ in inpatient and outpatient psychiatric care. Psychiatric inpatient care, often offered in locked hospital units providing both voluntary and involuntary care, includes nursing tasks like observation, taking responsibility for patients’ safety, stimulating patients to take part in social activities, administration of medication and administrative tasks (Frauenfelder, Muller‐Straub, Needham, & Achterberg, 2013; Mullen, 2009). In outpatient care, the nurses spend most of their time in counselling sessions with patients. As not all patients allow students to participate, it is a challenge for the preceptor to provide students with meaningful learning activities.

Nursing education in Sweden is comprised of a three‐year programme (180 credits) (SFS, 1993). The education is supposed to prepare the students to be capable of making clinical judgements independently and solving problems involving patient care, as well as keeping updated with the evidence‐based knowledge in nursing. The student should understand, identify and assess patients’ needs of care in somatic and psychiatric care. They should be prepared to organize, plan, lead, perform and evaluate nursing care and to supervise and educate staff. The nursing programme at Lund University focuses on person‐centred care and preparing nursing students to become lifelong learners in the nursing profession. During the second year, the students have five weeks of clinical training in either in‐ or outpatient psychiatric care, which is the one opportunity for nursing students to learn about psychiatric nursing. The public healthcare organization in southern Sweden has taken a joint decision with Lund University and the other two universities in the region (Malmö University and Kristianstad University) that PL should be the prioritized model in clinical nursing placements. The decision was based on the growing numbers of nursing students and reduced numbers of available clinical placements. The implementation of PL started in somatic inpatient settings. With the same backdrop of increasing numbers of students, reinforced by the fact that the psychiatric clinic had a shortage of available clinical placements, a rethinking the model of learning and teaching for nursing students at the psychiatric clinic was proposed. As psychiatric nursing includes more reflection, PL was viewed as an interesting model by the faculty.

In the spring of 2017, several workshops about PL were held by Lund University, followed by an organized implementation of PL at seven psychiatric units in the autumn of 2017. Preceptors were invited to meetings about PL to be prepared to act as preceptor for students with PL for the first time. Structured learning activities (SLAs) are written assignments and an important ingredient in PL which is intended to be used by peers as a learning tool. The context into which SLAs are introduced, as well as the expected learning outcomes, has to be considered (Boud et al., 2016). In the present study, teachers from the university together with the preceptors had composed twelve SLAs, related to the learning outcomes set by the university. Examples of SLAs are simulating taking a medical history from your peer and then completing the activity with a patient, observe your peer caring for a patient and reflect together about the patients' sense of security, describe somatic, psychological and psychosocial care offered to patients in psychiatric settings, identify routines that can be improved at the ward and find an article that support the suggestion. Each SLA requires actively inquiring and continuous reflection while the peers solve the assignment and afterwards with the preceptor.

As PL in psychiatric nursing had not been described earlier and was totally new for the psychiatric clinic in Lund, we decided to evaluate the experience. Data were collected during the first semester with PL at the six inpatient units and one outpatient unit who volunteered to try PL. To our knowledge, no previous studies included PL in outpatient care, neither in somatic nor psychiatric care. Therefore, the experience of PL at the only volunteering outpatient clinic was considered with an extra interest. The aim was to describe the experiences of PL in psychiatric inpatient settings during clinical placements for undergraduate nursing students from the preceptors’ and the students’ perspective and to highlight the possibility for PL in psychiatric outpatient settings.

## 3 DESIGN

A qualitative inductive design using a questionnaire and filmed interviews.

## 4 METHOD

A questionnaire was distributed to all preceptors and students in the six inpatient and the only outpatient psychiatric setting that had introduced PL the same semester. To further illuminate the experiences of PL in the outpatient setting, filmed interviews were conducted with one student and one preceptor at the first psychiatric outpatient clinic that had implemented PL. The purpose of the interviews was to get a deeper understanding of the experiences of PL, to give an example of how PL can be used at an outpatient unit and to include parts of the films in a presentation about PL in an outpatient psychiatric setting at a national nursing conference on clinical training (SSF, 2018).

### 4.1 Setting and participants

In the autumn semester 2017, 24 nursing students were placed at the seven psychiatric units that had volunteered to try PL for the first time. One of the units was an outpatient clinic. The students had been randomly paired and instructed to share clinical tasks, solve SLAs and reflect together and/or with the preceptor. Students could perform nursing tasks together or individually, with or without the preceptor, always with consideration for the patients’ needs and wishes. After their five weeks of psychiatric clinical training, all students were asked to complete a questionnaire and 14 students returned the questionnaire. The students were divided between two periods. All 25 preceptors who used PL in any of these periods were invited, and 12 of them returned the questionnaire. One month after the study period, the two students and the preceptor at the outpatient unit were asked to be interviewed and filmed and one student and the preceptor accepted.

### 4.2 Data collection

Two similar questionnaires (one for the students, one for the preceptors) were designed by KL. The first sentence was “Peer learning has recently been introduced at the unit where you are doing your clinical placement/have precepted nurse students. As part of the development of the clinical placements, please describe your experiences of peer learning by answering the questions below.” Ten questions, most of them open‐ended, followed. Examples of the questions are the following: “Have you and your peer/your students used the SLAs together? What is your experience of SLAs? To what extent did structured reflection between students/preceptor occur? How did you experience cooperation with your peer/between the students? Other suggestions/tips for the SLAs? In what situations did you/the students experience support from each other? Did peer learning bring advantages or disadvantages not related to learning?” Pre‐addressed envelopes with the questionnaires were distributed personally by a head preceptor during the third week of clinical placements. Informants were asked to fill in the questionnaire on the last day of the clinical placement. To maintain confidentiality, informants were not asked to write their names or which psychiatric unit the answers referred to.

The interviews with the preceptor and the student at the outpatient psychiatric clinic were filmed. The interviews, lasting for 40 and 55 min, included questions like those in the questionnaire.

### 4.3 Data analysis

The questions asking informants about tips for developing SLAs and other suggestions or comments were answered by half of the students and preceptors. The rest of the questions were answered by all informants, and many questions received a lengthy response. The first question—“What is your opinion of peer learning? Please describe advantages, disadvantages and challenges”—for example, rendered a mean 67 words. The handwritten answers in the questionnaires were transcribed in a Microsoft Word document. A content analysis on a manifest level was performed to categorize the comments written in the questionnaire (Kondracki, Wellman, & Amundson, 2002). The researcher examined the comments, noted applicable content categories and identified manifest content, that is visible or literally present content. Similar comments were grouped into categories derived from the data. Students’ and preceptors’ experiences are reported in each category.

The filmed interviews were transcribed. The text was coded and analysed (Kondracki et al., 2002) and compared with the results of the questionnaires.

### 4.4 Ethical considerations

Approval from an ethical board is not necessary for a course evaluation. Completion and return of the questionnaire were understood as consent to participate. The nurse and the student who participated in the filmed interviews signed informed consent and allowed the films to be used in presentations.

### 4.5 Trustworthiness

To ensure credibility, all students and all preceptors who had experience of PL were invited to participate. The interviews were performed two months after the clinical placement, allowing the participants time for reflection. At that time, the questionnaires had been analysed. Thus, probing questions mirroring the results of the questionnaires could be used in the interviews, to further explore experiences of PL. The analysis was conducted systematically at a manifest level, meaning that the visible content, literally present in the text, was analysed without interpretation. This is a suitable method when analysing data derived from a questionnaire where the content cannot reach the same depth as in an interview. The analysis was performed by the second author and verified by the first author. Quotations are presented to illustrate the categories.

## 5 RESULTS

The results showed that 13 out of 14 students were precepted by two preceptors, although one of the two preceptors supported both students during each working shift. The twelve preceptors had worked as registered nurses between one and 38 years and as a preceptor between one and 30 years. Five preceptors had previously precepted students with PL, and four preceptors had experience of PL as a nursing student. One student had previous experience of PL.

The results are presented in five categories: *learning through exchange of knowledge and reflection, collaboration created security and independence*, *structured learning activities (SLAs) as learning tools, learning opportunities and students’ compatibility*. The content of the interviews was consistent with the results of the questionnaire, although expressed with more words. The interviewed preceptor, who had previously used PL at inpatient units for many years, described how well PL also suited an outpatient unit where most of her time was spent counselling patients:

“It's all about preparations, collaboration and structure to meet the challenge to find meaningful activities for the students when patients don't want them to take part in counselling sessions. Actually, PL at an out‐patient clinic is even better, it allows students to systematically follow the entire nursing process in persons with psychiatric disease.” (Interviewed preceptor)

### 5.1 Learning through exchange of knowledge and reflection

Both students and preceptors recognized the benefits of PL for students’ learning. They described daily reflections and discussions between peers and between peers and preceptors regarding patient encounters and nursing tasks. These reflections were perceived as leading to personal development. Students appreciated seeking answers to their questions, not from their preceptor but together with their peer:

“I had the opportunity to reflect with my peer every day. We had reflection time with our preceptor before and after performing a challenging nursing task… These reflections were valuable and gave me a deeper insight into the situation we were discussing. I was presented with new perspectives on the situation.” (Student F)

“PL was beneficial for the students. They could help each other, learn from one another and by doing so, they learnt together. Students could discuss different patient cases and situations.” (Preceptor I)

Students discussed and planned nursing tasks together even though one of the students could have performed the task individually. Afterwards, the experience of the performed task was shared between the peers during continuous reflections. Hence, each student simultaneously learned and contributed to other students’ learning:

“Out of consideration for the patient, either I or my peer participated together with our preceptor in patient encounters. Afterwards we shared our experience with one another.” (Student E)

The interviewed preceptor pointed out that PL gave stimulation and personal development not only for students but also for the preceptors as they continuously had to reflect on their own nursing practice.

### 5.2 Collaboration created security and independence

Collaboration between students created a sense of security according to students and preceptors alike. Continuous support provided by the student peer and not feeling alone on the clinical placement encouraged students to confront challenges. Peers planned and equally divided the nursing tasks between themselves and provided support and space to one another. Students supported each other by being present through the performance of practical tasks. This was appreciated, especially during situations they found difficult or new and during patient encounters when the student felt unconfident and hesitant to do the task individually.

Students’ collaboration was encouraged and supported by preceptors. When the preceptor encouraged the students to work together, PL was perceived to facilitate students in being active participants in the nursing tasks and making students feel confident:

“… They had each other to consult with. In my experience students were more independent than they usually are while working with patients.” (Preceptor E)

“The feeling of not being the only student at my clinical placement, of being alone, gave me the courage to take on the nursing tasks and therefore I learnt more. I felt we were allowed to perform tasks since we were two students.” (Student D)

### 5.3 Structured learning activities (SLAs) as a learning tool

Both students and preceptors were positive about the SLAs, which was perceived to encourage discussions and reflections and exchange of new perspectives between peers. The link between theory and practice in psychiatric care became evident:

“SLAs provided support and guidance in my learning at times when I was not occupied with the nursing tasks. SLAs helped me apprehend the link between theoretical knowledge and practical tasks. I did the structured learning activities with my peer mostly but also on my own." (Student N)

When the preceptor was occupied with tasks that the student peers could not take part in, the peers could work together with the SLAs. The interviewed preceptor pointed out that this opportunity reduced the stress on the preceptor: she could rely on the SLAs to give the students a meaningful learning experience even when they could not participate in the counselling sessions. The SLAs were suitable tasks for students to perform to gain more knowledge. With PL the students were perceived to become more independent, they reflected and solved problems together and discussed what was still unsolved with the preceptor, which saved her time.

### 5.4 Learning opportunities

Fewer opportunities for training medical–technical skills compared with earlier clinical training in somatic care were recognized as a disadvantage by some students and preceptors as they had to divide the patient‐related tasks between them. The peers also needed to share the time spent with the preceptor. This was mentioned as a potentially missed learning opportunity for each student:

“…I got less time with my preceptor and fewer patient encounters. In my experience I got fewer opportunities for training in technical skills. There was a risk that I missed out an interesting experience because my peer did the task.” (Student I)

When preceptors were engaged in activities that students could not take part in, some students were disappointed. On the other hand, the interviewed student described how she and her peer could use time that otherwise would have been downtime. Besides the SLAs, they did roleplay and found that valuable. At the outpatient clinic, only one of the students could participate with the patient and the preceptor at each counselling session. However, the interviewed student did not recognize this as a loss of meaningful activities. She put it like this:

“We prepared ourselves by reading the medical record. Then one of us participated in the counselling hour with the patient and our preceptor. Afterwards my peer and I sat down and reflected: what had happened, how the nurse handled the situation, what could have been done in another way. So, we participated in half as many counselling hours but instead got double reflection…” (Interviewed student)

### 5.5 Students’ compatibility

A few students and preceptors experienced students not being compatible on personal and professional levels. A self‐confident student could appear to take the lead in discussions and reflections besides performing nursing tasks that suited the student, without consideration for the other student. This could create insecurity for the other student, who stepped back due to a feeling of competition between the students:

“…My peer took a lot of space. I am more cautious and I felt like I didn't get any space. I felt a bit invaded by my peer…My peer didn't seem encouraged to collaborate with me. It felt like she was challenging me about who did more nursing tasks and who was more independent.” (Student K)

Preceptors tackled situations with peers who were incompatible through discussions with both students to enable equal learning assignments for the students:

“The imbalance between students was corrected after two weeks. Both students were content at the end of their clinical placement…” (Preceptor K)

## 6 DISCUSSION

Most students and preceptors in the present evaluation valued the perceived learning benefits of PL. Reflection and discussion between peers and preceptors were perceived to contribute to a deeper understanding of psychiatric nursing. Furthermore, peer collaboration and use of SLAs was perceived to contribute to a secure learning atmosphere and increased the students’ self‐confidence. Hence, PL contributed to students’ professional development. These results are in line with previous research on PL for nursing students in somatic care (Chojecki et al., 2010; Hellström‐Hyson et al., 2012; Mamhidir et al., 2014; Stenberg & Carlson, 2015) and in psychiatric care (Vuckovic et al., 2019).

Both students and preceptors in the present evaluation emphasized how PL gave many opportunities for reflection. This is interesting in the light of previous studies discussing the importance of reflection as a learning strategy to facilitate nurse students’ learning in a psychiatric context (Holst & Hörberg, 2012; Hwang et al., 2018; Vuckovic et al., 2019). The present evaluation, to our knowledge the first report on PL in an outpatient setting, also shows a possibility and even advantages of using PL in outpatient clinics, where precepting students may bring other challenges compared with inpatient settings. With increasing numbers of students, fewer psychiatric wards and a lack of preceptors the possibility of implementing PL in outpatient settings is interesting. The novelty of PL in outpatient settings makes the present evaluation interesting even with a small number of participants.

The SLAs guided students towards an understanding of issues specific for psychiatric nursing and of what was expected of them during the clinical placement in psychiatry, contributing to a secure learning environment for the students as suggested by Stenberg and Carlson (2015). Additionally, solving SLAs without the preceptor's presence permitted students to work independently and was timesaving for the preceptor, which is consistent with the recent study by Stenberg, Bengtsson, Mangrio, and Carlson (2020). Problem‐based activities like SLAs with continuous opportunity for reflection promote a scientific approach to knowledge in both theory and practice, and we recommend such activities as a pedagogical tool during clinical placements.

Most preceptors and students in the present study had no previous experience of PL, so the concept of PL based on active students’ participation and learning from each other was unfamiliar to them. As students can benefit from PL provided that guidelines are clear and students are prepared (Boud et al., 2016), it is essential that enough information and preparation for PL are provided prior to clinical placements for students and preceptors alike (Nygren & Carlson, 2017; Stenberg & Carlson, 2015).

Students’ incompatibility was revealed in the present study as challenging for a few students, creating insecurity and competition. On the other hand, incompatible peers were recognized as an opportunity for teamwork training and preparation for the future preceptorship role for the students. Preceptors described how they had adjusted the teaching in discussions with the students. This contrasts with previous research where preceptors switched to traditional supervision when the students were incompatible (Mamhidir et al., 2014). It could be that preceptors in the present study created an understanding relationship with the peers and dared to address the challenge of students’ incompatibility and by doing so allowed an individual learning process for each of the students. This is important as the student's opportunity for learning during clinical placements in psychiatric contexts depends largely on the relationship with the preceptor (Happell et al., 2015). The preceptor's approach and way of communicating could influence students’ learning (Sundler et al., 2014).

Preceptors in the present study did not perceive PL as time consuming and stressful, as found in previous research (Nygren & Carlson, 2017). It could be that the structure of PL in the present evaluation, with two preceptors who were alternately responsible for the two students, facilitated the preceptor role. With this interpretation of PL, the pair of students could have different preceptors from one day to another but the students were supposed to always work as peers. Allowing two preceptors to alternate as supervisors for each pair of students did not follow the original intention and was surprising for the faculty, as the clinic's shortage of preceptors was one of the reasons for the joint decision between the clinic and the faculty to implement PL. This possibility between preceptors to collaborate with a pair of students might have been decisive for the seven units that volunteered to try PL. Support and feedback, as well as being able to plan and prepare for the clinical placement period, can be helpful for preceptors’ performance (Mårtensson, Engström, Mamhidir, & Kristofferzon, 2013). Another study (Ekstedt et al., 2019) indicated that students experienced greater advantages from having more than one preceptor compared with students with one preceptor while using PL but this model of PL is not useful if the reason for the implementation of PL is a lack of preceptors.

Security in peer students and in supervision contributed to the learning process for the students in psychiatric inpatient care when all aspects of the supervision were functioning, but it was burdensome when it did not work. Lack of cooperation between the pair of students, besides lack of reflections supported by the supervisor, could impair and hinder the learning process for the students (Holst & Hörberg, 2012). Another qualitative evaluation of a similar model, coaching and peer‐assisted learning for mental health nursing students, revealed overall positive students’ experiences, although the positive outcomes were dependent on the quality of peer support between mental health nursing students who were on different levels of nursing education (Wareing et al., 2018).

## 7 IMPLICATIONS FOR PRACTICE

In conclusion, the findings indicate that PL could contribute to nursing students’ learning in psychiatric inpatient care and in a psychiatric outpatient care. The extended discussions and reflections that PL offer, together with SLAs, may enhance reciprocal learning. Both theoretical and clinical education should be based on evidence and stimulate reflection and knowledge seeking for nursing students, which successively expands students’ knowledge acquisition. Through reflection on practice, which PL promotes, nursing students could be prepared for their future profession and hopefully continue to use reflection as a learning tool for their further professional development (Pålsson, 2020) and for their future role as preceptors. Thus, we recommend PL for nursing students not only in somatic settings but also in psychiatric settings.

To ensure the quality of PL, the teachers at the university and teachers employed at the clinics should, in collaboration, provide an introduction to PL for students, preceptors and staff. When the concept of PL is clear for all involved, the student's competence in psychiatric nursing, teamwork and preceptorship skills could benefit. Future research should focus on comparison of students’ learning with different preceptorship models and on identifying the necessary strategies for handling incompatible students when using PL. In‐depth interviews and focus groups will give a deeper understanding of students’ and preceptors’ experiences and are recommended in future research. Another topic for further research is the patients’ experience of being cared for by nursing students who are precepted by PL.

## 8 STRENGTHS AND LIMITATIONS

The data derived from the two interviews provided a deeper understanding of PL in an outpatient setting and corresponded with the data from the questionnaires, which is a strength. Forty‐eight per cent of the preceptors and 58% of the students who had tried PL answered the questionnaire at the end of the course. Although this is a higher percentage than the 19% of all 74 students in the course who evaluated the course in the standard electronic questionnaire at the end of the study period, the small sample limits the generalizability of the results. The analysis was performed by both authors separately and discussed until consensus was reached. We adhered to the COREQ guidelines.

## Conflict of Interest

The authors have no conflict of interest to declare.

## Author Contributions

KL planned the study and conducted the interviews. Both authors participated actively in the analysis and in writing the paper.

## ETHICAL CONSIDERATIONS

Approvement from an ethical board is not necessary for a course evaluation. Informants were informed that the aim was to evaluate the new preceptor model of peer learning and that the survey answers should be analysed and used in a pedagogical report. Completion and return of the questionnaire were understood as consent to participate. The nurse and the student who participated in the filmed interviews signed informed consent and allowed the films to be used in presentations.
